# Prediction models for refractory *Mycoplasma pneumoniae* pneumonia and corticosteroid treatment escalation in hospitalized children: a retrospective cohort study

**DOI:** 10.3389/fped.2026.1913556

**Published:** 2026-09-21

**Authors:** Yueqian Li, Yinan Shen, Xiao Shi, Yanwei Li, Xuan Li, Junfeng Liu

**Affiliations:** Third Department of Pediatrics, Handan First Hospital, Handan, Hebei, China

**Keywords:** children, corticosteroid escalation, glucocorticoid, *Mycoplasma pneumoniae* pneumonia, refractory MPP

## Abstract

**Background:**

*Mycoplasma pneumoniae* carries a clinically important risk of a refractory course or the need for intensified corticosteroid therapy. No admission-day prediction tool has been validated to identify children at risk for either outcome.

**Methods:**

Children admitted to a single tertiary centre with a diagnosis of MPP between January 2022 and June 2025 were enrolled in a retrospective cohort study. Two penalised logistic regression models were constructed from routine admission-day clinical and laboratory variables: one targeting RMPP occurrence across the full eligible cohort (Cohort A) and one targeting corticosteroid escalation within the treated subgroup (Cohort B). Candidate predictors were screened by LASSO-penalised regression, and model performance was optimism-corrected via bootstrap resampling. Discrimination, calibration, and clinical utility were assessed by corrected AUC, calibration metrics, and decision curve analysis, with nomograms derived from the final coefficients.

**Results:**

Of 383 Cohort A patients, 106 met criteria for RMPP. Within Cohort B (*n* = 197), 61 children (31.0%) required treatment escalation. The RMPP model retained nine admission-day variables, including pre-admission fever duration, log-transformed CRP, LDH, D-dimer, ferritin, the neutrophil-to-lymphocyte ratio, albumin, male sex, and pleural effusion, and yielded an optimism-corrected AUC of 0.847 (95% CI: 0.810–0.886). The escalation model retained four variables (LDH, NLR, ferritin, and CRP), with a corrected AUC of 0.799 (95% CI: 0.747–0.861). Both models achieved good calibration and positive net clinical benefit across decision thresholds from 5% to 60%.

**Conclusions:**

Our study offers a quantitative framework for stratifying children with MPP by their risk of refractory disease and treatment intensification.

## 1 Introduction

As a leading aetiological agent of paediatric CAP, *Mycoplasma pneumoniae* (M. pneumoniae) disproportionately affects school-age children and is estimated to account for 10%–40% of CAP hospitalisations in this age group (1). A pronounced global resurgence of M. pneumoniae infections occurred in 2023 in the wake of the COVID-19 pandemic. In China, this surge was driven predominantly by the widespread dissemination of macrolide-resistant strains (2), a phenomenon further amplified by the accumulation of susceptibility attributable to pandemic-era immune debt (3). Although MPP follows a benign, self-resolving trajectory in the majority of cases, a clinically important minority of children develop sustained pyrexia and progressive radiographic deterioration despite appropriate antimicrobial treatment, culminating in refractory MPP (RMPP). This condition is associated with substantial risks of pulmonary sequelae and extrapulmonary organ involvement (4), and timely identification of high-risk individuals is therefore essential for tailoring surveillance intensity and the initiation of adjunctive therapies.

Considerable evidence has accumulated regarding disease-severity biomarkers relevant to RMPP. Pooled data from meta-analyses indicate that affected children exhibit markedly elevated serum concentrations of LDH, CRP, neutrophil fraction, interleukin-6, and erythrocyte sedimentation rate relative to non-RMPP counterparts, with lobar consolidation and parapneumonic pleural effusion identified as significant radiological risk factors (5). At the individual-marker level, pre-admission fever persisting beyond 10 days, a CRP concentration exceeding 40 mg/L, and consolidation involving at least two-thirds of a pulmonary lobe have each demonstrated independent prognostic relevance (6). Multivariable modelling further established D-dimer, CRP, and LDH as independent predictors, each attaining receiver operating characteristic AUC values above 0.84 (7). These observations have informed the construction of nomograms and composite scoring instruments for early prognostication (8, 9). Notwithstanding these advances, the majority of existing tools were developed without correction for model optimism, no validated multivariable composite model integrating multiple admission-day variables has been developed specifically for treatment escalation prediction, and comparatively little attention has been directed toward guiding subsequent therapeutic decisions. Corticosteroids constitute the principal immunomodulatory treatment for RMPP and are conventionally initiated at methylprednisolone 2 mg/kg/day, with escalation to higher-dose or pulse regimens reserved for cases that fail to respond, although optimal escalation criteria remain a matter of ongoing debate (10, 11). When an adequate response to standard-dose therapy is not achieved, intensification through dose escalation or the addition of intravenous immunoglobulin may become necessary (12), yet no validated tool currently exists to anticipate this need at the time of admission.

There, two parallel penalised logistic regression models were developed. The first model estimated the probability of RMPP in the complete eligible population. The second was restricted to the subgroup receiving systemic corticosteroids and targeted the likelihood of requiring treatment escalation. Predictor selection employed LASSO regularisation, optimism correction was achieved through bootstrap resampling, and decision curve analysis alongside nomogram construction was performed to facilitate practical clinical implementation.

## 2 Materials and methods

### 2.1 Study population

Children hospitalised with MPP at Handan First Hospital between January 2022 and June 2025 were enrolled in a single-centre retrospective cohort study. Eligibility required fulfilment of three criteria (Supplementary Figure S1). An MPP diagnosis conforming to the Chinese Guidelines for the Diagnosis and Treatment of Mycoplasma pneumoniae Pneumonia in Children (2023 edition) was required, established by a positive M. pneumoniae nucleic acid test and/or a serum antibody titre of 1:160 or greater, or a fourfold rise between paired samples (12). Eligible children were also required to be between 28 days and 18 years of age, with complete core admission data available. Children were excluded if any of the following applied: (i) the clinical presentation was primarily attributable to a co-infecting pathogen rather than M. pneumoniae; (ii) a pre-existing diagnosis of primary immunodeficiency, malignancy, haemodynamically significant congenital heart disease, or chronic lung disease; (iii) ongoing long-term systemic corticosteroid or immunosuppressive therapy; (iv) initiation of corticosteroids or escalated treatment before admission; (v) an indeterminate outcome owing to early discharge, inter-facility transfer, or loss to follow-up; or (vi) absence of one or more core admission variables.

Two cohorts were constituted from the eligible population. Cohort A encompassed all eligible children with MPP and provided the development dataset for the RMPP prediction model. Cohort B was a nested subset restricted to those who received standard-dose systemic corticosteroids (methylprednisolone 1–2 mg/kg/day or equivalent) during the index hospitalisation, with doses from heterogeneous regimens converted to methylprednisolone-equivalent quantities using published conversion factors as specified in the Clinical Application Guidelines for Corticosteroids (2023 edition) (13).

### 2.2 Outcome definitions

RMPP (Cohort A) was operationally defined as pyrexia persisting beyond 72 h in spite of guideline-recommended macrolide antibiotic therapy, alongside clinical or radiographic worsening in at least one domain (10, 12).

Corticosteroid escalation (Cohort B) was considered to have occurred when, after a minimum of 24 to 48 h of standard-dose methylprednisolone (approximately 1–2 mg/kg/day) and in the context of an inadequate treatment response, management was advanced to a high-dose regimen (methylprednisolone ≥4 mg/kg/day), pulse-dose methylprednisolone (≥10 mg/kg/day for three consecutive days), or addition of intravenous immunoglobulin (IVIG) (13). An inadequate treatment response was defined as a decrease in peak body temperature of less than 1 °C from the pre-treatment value at 24 to 48 h after initiating standard-dose corticosteroid therapy, in the absence of appreciable improvement in either clinical symptoms or chest imaging appearances.

### 2.3 Candidate predictors

Fourteen candidate predictors were pre-specified before model construction. Of these, eleven were continuous measures, comprising age in months, pre-admission fever duration in days, CRP (mg/L), LDH (U/L), D-dimer (mg/L), ferritin (ng/mL), NLR, albumin (g/L), platelet count (×10⁹/L), fibrinogen (g/L), and CK-MB (U/L). The remaining three were binary variables, encompassing male sex, pulmonary consolidation spanning at least two-thirds of a lobe, and pleural effusion.

### 2.4 Statistical analysis

Continuous variables exhibiting non-normal distributions are reported as medians with interquartile ranges, and between-group comparisons were performed using the Mann–Whitney U test. Categorical data are summarised as counts with proportions, and group differences were evaluated with the chi-square test.

Predictor selection employed LASSO-penalised regression applied across all 14 pre-specified candidates (14). The regularisation parameter *λ* was tuned by five-fold cross-validation, with the one-standard-error rule (*λ*.1se) preferred to promote parsimony and reduce overfitting. The minimum cross-validation error criterion (*λ*.min) served as a fallback whenever fewer than two variables survived the primary selection step.

Final penalised logistic regression models were fitted using the lrm function within the rms package in R, incorporating the LASSO-retained variables as covariates for each outcome separately. Associations are quantified as odds ratios with 95% confidence intervals, with continuous predictors scaled to a one-standard-deviation increment and binary predictors referenced against the unexposed category.

Optimism correction was achieved through bootstrap resampling (B = 2,000), in which the complete LASSO selection and model-fitting sequence was replicated within each resample. The resulting optimism estimate was subtracted from apparent metrics to produce corrected concordance statistics with 95% confidence intervals and corrected calibration parameters. Five-fold cross-validation was used to generate a supplementary AUC estimate. Model calibration was examined visually through locally weighted (loess) smoothed calibration curves and quantified using three numerical indices, comprising the integrated calibration index (ICI), the median absolute calibration error (E50), and the 90th-percentile absolute calibration error (E90). Overall predictive accuracy was further characterised by the Brier score. The clinical value of each model was examined through decision curve analysis (DCA), quantifying net benefit across threshold probabilities from 5% to 60% and benchmarking against universal-treatment and no-treatment reference strategies. Point-based nomograms were derived from the final model coefficients, with all predictor scales expressed in their original clinical measurement units. All statistical analyses were carried out in R (version 4.4.2), and a two-tailed *P*-value of less than 0.05 was adopted as the threshold for statistical significance.

## 3 Results

### 3.1 Study cohort and baseline characteristics

A total of 383 children with MPP were enrolled over the study period (Supplementary Table S1). The median age was 6.5 years, and 190 patients (49.6%) were male. RMPP occurred in 106 children, corresponding to an incidence of 27.7%. Among 197 children who received systemic corticosteroids, 61 (31.0%) underwent treatment escalation.

Compared with children without RMPP, those in the RMPP group had significantly more days of fever before admission, a longer overall illness duration, higher peak temperature, and elevated levels of neutrophil percentage, NLR, CRP, procalcitonin, erythrocyte sedimentation rate, LDH, ferritin, D-dimer, CK-MB, and IL-6 (*P* < 0.05), alongside lower serum albumin (*P* < 0.001) (Table 1). Radiographically, the prevalence of pulmonary consolidation, extensive consolidation involving ≥2/3 of a lobe, and pleural effusion was higher in the RMPP group (*P* < 0.05). Age, sex, white blood cell count, platelet count, hemoglobin, fibrinogen, and alanine aminotransferase (ALT) did not differ significantly between groups (*P* > 0.05).

**Table 1:** Baseline characteristics of the study cohort stratified by RMPP status.

Characteristic	Overall (*n* = 383)	Non-RMPP (*n* = 277)	RMPP (*n* = 106)	*P*-value
Demographics				
Age, months	78.0 [54.0, 98.0]	79.0 [56.0, 98.0]	77.5 [49.0, 95.8]	0.563
Male sex, *n* (%)	190 (49.6)	146 (52.7)	44 (41.5)	0.065
Clinical features				
Fever duration, days	5.0 [3.0, 7.0]	4.0 [3.0, 6.0]	6.0 [4.0, 9.0]	<0.001
Illness duration, days	6.0 [5.0, 8.5]	6.0 [5.0, 8.0]	8.0 [6.0, 10.0]	<0.001
Peak temperature, °C	39.1 [38.6, 39.5]	39.0 [38.6, 39.3]	39.3 [38.8, 39.7]	<0.001
Prior macrolide, *n* (%)	200 (52.2)	149 (53.8)	51 (48.1)	0.378
Asthma/atopy, *n* (%)	75 (19.6)	55 (19.9)	20 (18.9)	0.941
Wheeze, *n* (%)	93 (24.3)	70 (25.3)	23 (21.7)	0.551
Dyspnea, *n* (%)	82 (21.4)	55 (19.9)	27 (25.5)	0.289
Oxygen at admission, *n* (%)	25 (6.5)	18 (6.5)	7 (6.6)	1.000
Laboratory findings				
WBC, G/L	8.5 [6.3, 10.6]	8.3 [6.2, 10.5]	9.0 [6.7, 10.7]	0.182
Neutrophils, %	62.8 [55.7, 69.3]	61.3 [53.1, 67.0]	67.0 [60.9, 75.7]	<0.001
NLR	2.2 [1.7, 2.9]	2.0 [1.6, 2.5]	2.8 [2.1, 3.6]	<0.001
Platelet count, G/L	305.0 [240.5, 366.5]	305.0 [239.0, 376.0]	306.5 [247.8, 359.0]	0.775
Hemoglobin, g/L	119.0 [112.0, 126.0]	120.0 [113.0, 128.0]	118.0 [111.2, 124.8]	0.062
CRP, mg/L	19.0 [9.6, 33.5]	15.4 [8.0, 26.1]	31.4 [19.6, 49.9]	<0.001
Procalcitonin, ng/mL	0.3 [0.1, 0.5]	0.2 [0.1, 0.5]	0.4 [0.2, 0.6]	0.001
ESR, mm/h	36.0 [22.2, 50.0]	35.5 [21.0, 49.0]	39.5 [28.0, 53.2]	0.040
LDH, U/L	323.0 [223.5, 447.0]	283.0 [200.0, 392.0]	447.5 [327.0, 688.2]	<0.001
Ferritin, ng/mL	138.0 [80.0, 252.5]	118.0 [67.0, 203.0]	231.5 [135.5, 412.8]	<0.001
D-dimer, mg/L	0.7 [0.4, 1.4]	0.6 [0.3, 1.1]	1.3 [0.6, 2.7]	<0.001
Fibrinogen, g/L	4.2 [3.5, 4.9]	4.1 [3.5, 4.9]	4.3 [3.8, 4.9]	0.087
ALT, U/L	20.0 [9.5, 38.5]	20.0 [10.0, 34.0]	20.5 [9.0, 47.0]	0.634
Albumin, g/L	40.0 [37.4, 43.0]	40.6 [38.0, 43.5]	38.8 [36.0, 40.6]	<0.001
CK-MB, U/L	21.0 [14.0, 28.0]	21.0 [14.0, 27.0]	23.5 [17.0, 30.0]	0.013
IL-6, pg/mL	12.3 [6.5, 25.0]	10.7 [6.0, 20.2]	20.3 [9.3, 37.8]	<0.001
Radiological findings				
Consolidation (any), *n* (%)	239 (62.4)	160 (57.8)	79 (74.5)	0.004
Consolidation ≥2/3 lobe, *n* (%)	64 (16.7)	37 (13.4)	27 (25.5)	0.007
Pleural effusion, *n* (%)	63 (16.4)	30 (10.8)	33 (31.1)	<0.001
Atelectasis, *n* (%)	51 (13.3)	38 (13.7)	13 (12.3)	0.836

Continuous variables are summarised as median [interquartile range] and compared with the Mann–Whitney U test; categorical variables are summarised as count (percentage) and compared with the chi-square test.

ALT, alanine aminotransferase; CK-MB, creatine kinase MB isoenzyme; CRP, C-reactive protein; ESR, erythrocyte sedimentation rate; IL-6, interleukin-6; LDH, lactate dehydrogenase; NLR, neutrophil-to-lymphocyte ratio; RMPP, refractory Mycoplasma pneumoniae pneumonia; WBC, white blood cell count.

Baseline comparisons between the escalation and non-escalation subgroups (Supplementary Table S1) showed that children who required escalation had significantly higher values for days of fever before admission, illness duration, neutrophil percentage, NLR, CRP, procalcitonin, LDH, ferritin, D-dimer, ALT, and IL-6 (*P* < 0.05), as well as lower albumin and hemoglobin concentrations (*P* < 0.05). Age, sex, and peak temperature did not differ significantly between the two subgroups.

### 3.2 LASSO variable selection

For the RMPP model, LASSO regression under the *λ*.1se criterion selected 9 of the 14 candidate variables (Supplementary Table S2): days of fever before admission, log-transformed CRP, log-transformed LDH, log-transformed D-dimer, log-transformed ferritin, NLR, albumin, male sex, and pleural effusion. The events-per-variable ratio (EPV) was 11.8, satisfying the recommended minimum threshold of 10.

For the corticosteroid escalation model, the same criterion retained 4 variables (Supplementary Table S2): log-transformed CRP, log-transformed LDH, log-transformed ferritin, and NLR. The EPV was 15.2.

### 3.3 RMPP prediction model

The nine LASSO-selected variables were entered into a penalized logistic regression model (Table 2, Figure 1D), with effect sizes for continuous predictors expressed per one standard deviation increase. Days of fever before admission (OR = 1.93, 95% CI: 1.43–2.59, *P* < 0.001), CRP (OR = 1.88, 95% CI: 1.34–2.63, *P* < 0.001), LDH (OR = 1.73, 95% CI: 1.22–2.44, *P* = 0.002), D-dimer (OR = 1.57, 95% CI: 1.14–2.15, *P* = 0.005), and ferritin (OR = 1.42, 95% CI: 1.03–1.95, *P* = 0.032), with the latter four entered on the log scale, were each independently associated with an increased risk of RMPP. Pleural effusion was also a significant predictor (OR = 2.08, 95% CI: 1.03–4.22, *P* = 0.042). Male sex was associated with a 51% reduction in the odds of RMPP relative to female sex (OR = 0.49, 95% CI: 0.28–0.88, *P* = 0.018). NLR and albumin were retained in the model through LASSO selection but did not achieve statistical significance (OR = 1.26, *P* = 0.184 and OR = 0.88, *P* = 0.426, respectively).

**Table 2:** Multivariable logistic regression model for RMPP and corticosteroid escalation.

Predictor	Unit	Odds ratio (95% CI)	*P*-value
RMPP			
Fever duration	per 1 SD	1.93 (1.43–2.59)	<0.001
CRP (log)	per 1 SD	1.88 (1.34–2.63)	<0.001
LDH (log)	per 1 SD	1.73 (1.22–2.44)	0.002
D-dimer (log)	per 1 SD	1.57 (1.14–2.15)	0.005
Ferritin (log)	per 1 SD	1.42 (1.03–1.95)	0.032
NLR	per 1 SD	1.26 (0.90–1.76)	0.184
Albumin	per 1 SD	0.88 (0.64–1.21)	0.426
Male sex	yes vs. no	0.49 (0.28–0.88)	0.018
Pleural effusion	yes vs. no	2.08 (1.03–4.22)	0.042
Corticosteroid escalation			
CRP (log)	per 1 SD	1.54 (1.02–2.34)	0.042
LDH (log)	per 1 SD	2.23 (1.44–3.44)	<0.001
Ferritin (log)	per 1 SD	1.66 (1.10–2.50)	0.015
NLR	per 1 SD	2.05 (1.10–3.79)	0.023

Estimates are from the multivariable logistic model fitted to the predictors retained by LASSO. For continuous predictors the odds ratio refers to a 1-standard-deviation increment; CRP, LDH, D-dimer and ferritin were natural-log transformed before modelling.

RMPP, refractory Mycoplasma pneumoniae pneumonia; CI, confidence interval; CRP, C-reactive protein; LASSO, least absolute shrinkage and selection operator; LDH, lactate dehydrogenase; NLR, neutrophil-to-lymphocyte ratio; SD, standard deviation.

**Figure 1:**
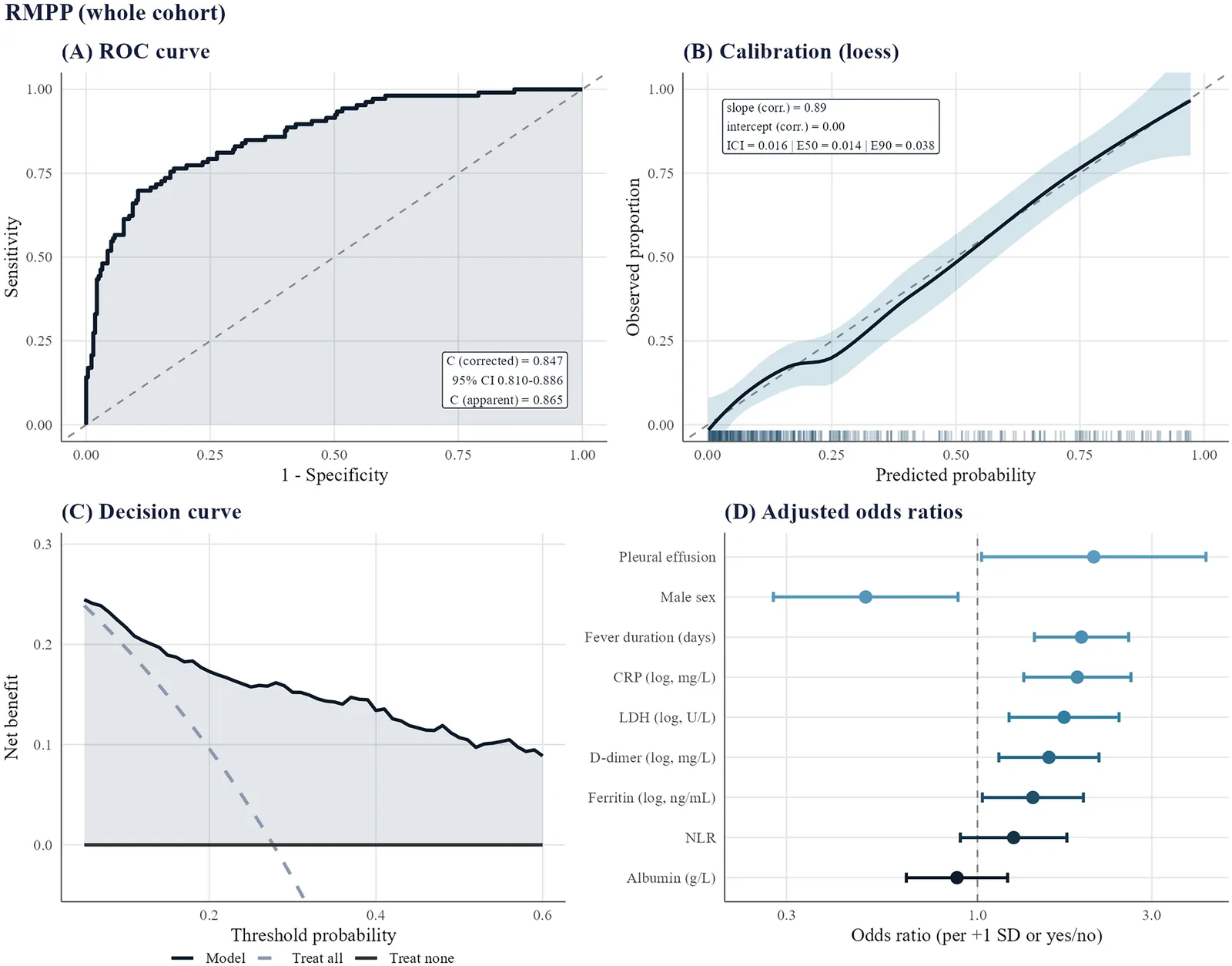
Development and internal validation of the RMPP prediction model. **(A)** ROC curve, with the inset reporting the apparent and bootstrap optimism-corrected concordance statistics with 95% CI. **(B)** Loess-smoothed calibration curve, with the inset reporting the corrected calibration slope and intercept, ICI, E50, and E90. **(C)** Decision curve analysis. **(D)** Forest plot of adjusted odds ratios with 95% CIs.

The apparent AUC was 0.865. Following optimism correction via bootstrap resampling (B = 2,000), the corrected AUC was 0.847 (95% CI: 0.810–0.886), reflecting an estimated optimism of 0.018 (Figure 1A). Calibration metrics indicated close agreement between predicted and observed probabilities, with a corrected slope of 0.89, an intercept of 0.00, ICI = 0.016, E50 = 0.014, and E90 = 0.038 (Supplementary Table S3, Figure 1B). The loess-smoothed calibration curve closely approximated the ideal diagonal. The Brier score was 0.122. Decision curve analysis demonstrated that model-guided classification conferred net benefit over both the treat-all and treat-none reference strategies across threshold probabilities from 5% to 60% (Figure 1C). A nomogram derived from the final model coefficients is presented in Figure 2A, with all predictor axes displayed in their original measurement units (Supplementary Table S4).

**Figure 2:**
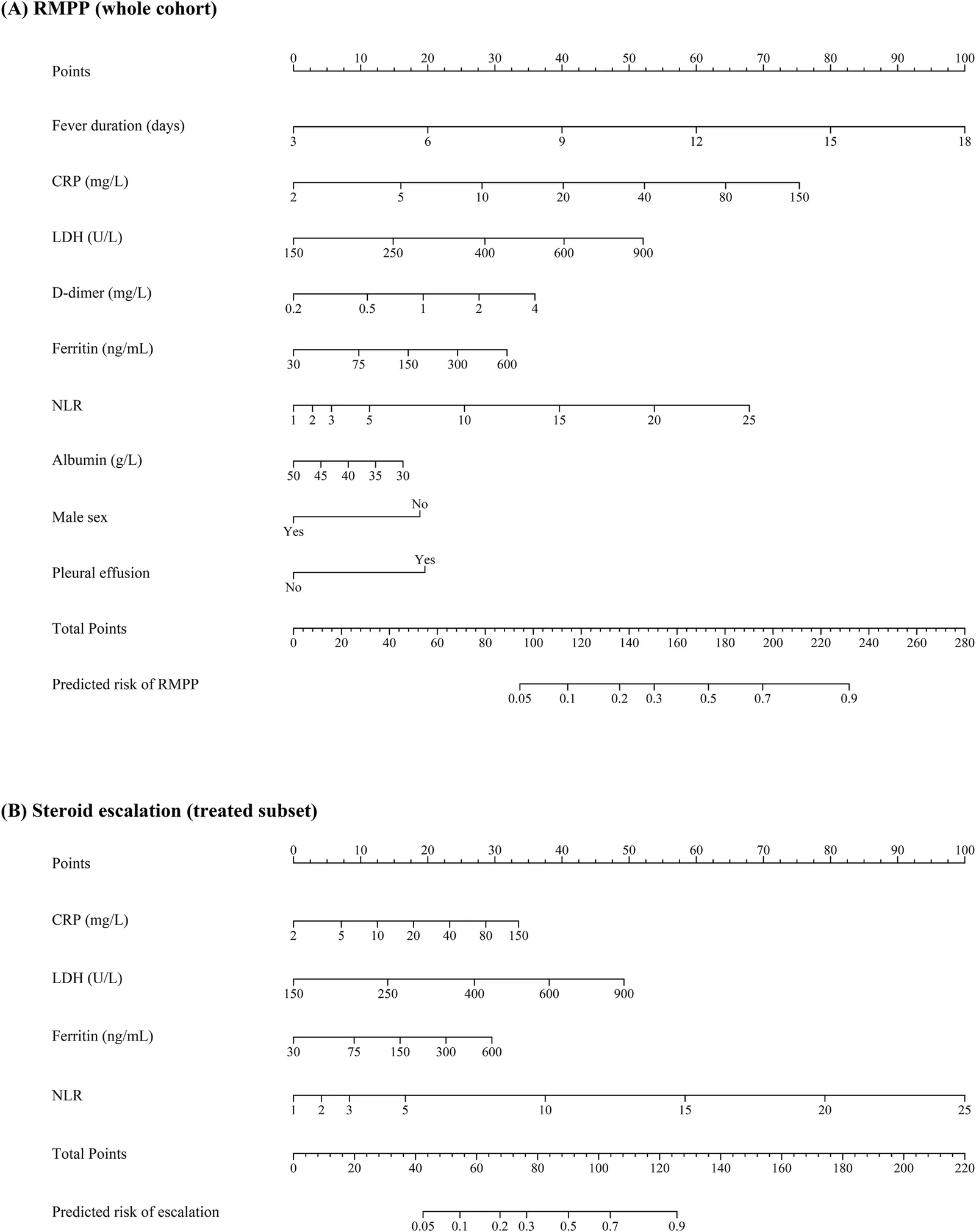
Nomograms for individualised risk estimation. **(A)** Nomogram for predicting the probability of RMPP. **(B)** Nomogram for predicting the probability of corticosteroid escalation among treated children. Predictor axes are displayed on the original measurement scale, with CRP, LDH, D-dimer, and ferritin modelled internally on the natural-log scale.

### 3.4 Corticosteroid escalation prediction model

The four LASSO-selected variables were incorporated into a separate penalized logistic regression model (Table 2, Figure 3D), with continuous predictors again expressed per one standard deviation increase. LDH (OR = 2.23, 95% CI: 1.44–3.44, *P* < 0.001), NLR (OR = 2.05, 95% CI: 1.10–3.79, *P* = 0.023), ferritin (OR = 1.66, 95% CI: 1.10–2.50, *P* = 0.015), and CRP (OR = 1.54, 95% CI: 1.02–2.34, *P* = 0.042), with LDH, ferritin, and CRP entered on the log scale, were all independently associated with the occurrence of corticosteroid escalation.

**Figure 3:**
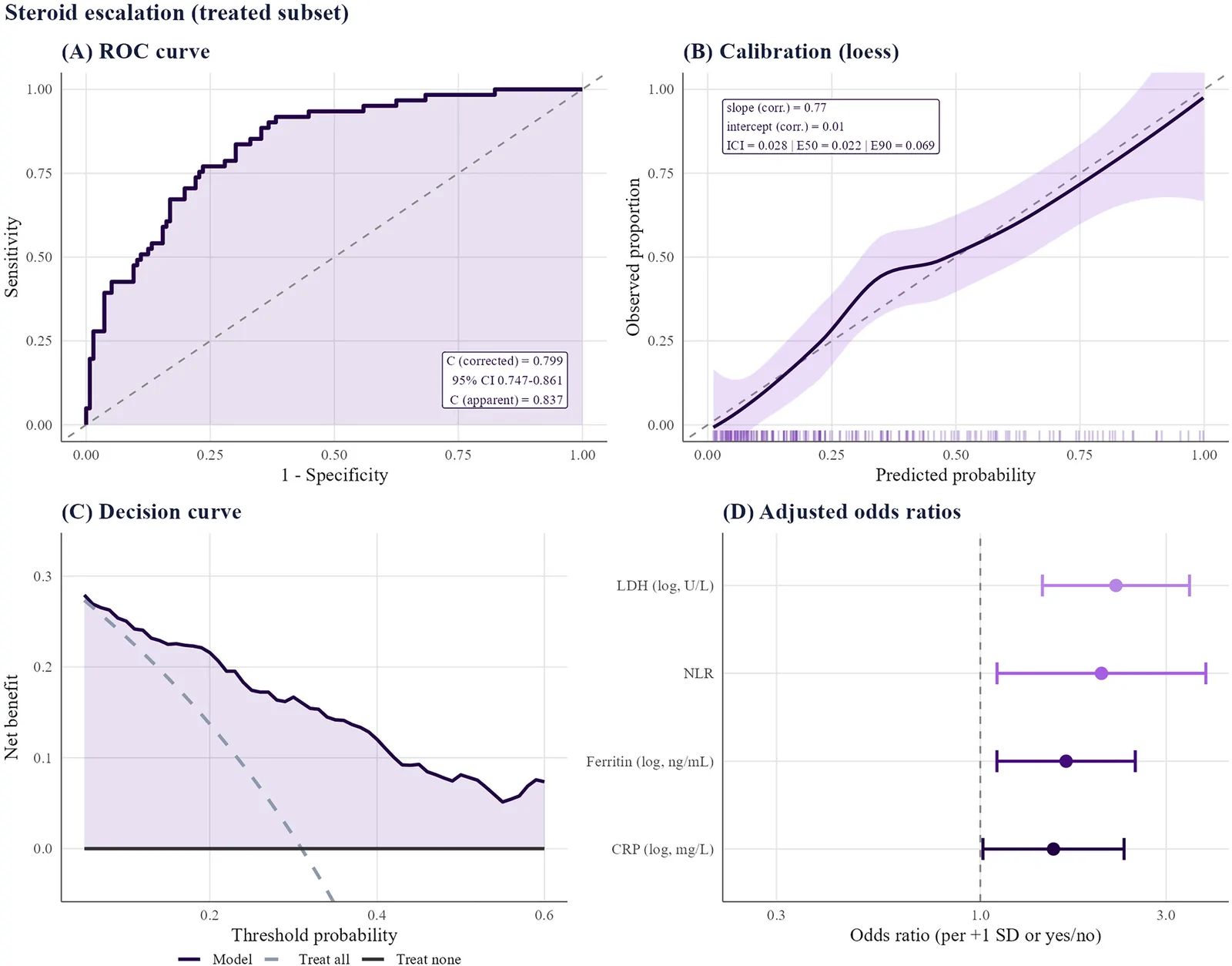
Development and internal validation of the corticosteroid escalation prediction model. **(A)** ROC curve with the apparent and optimism-corrected concordance statistics. **(B)** Loess-smoothed calibration curve with the corrected calibration slope, intercept, ICI, E50, and E90. **(C)** Decision curve analysis. **(D)** Forest plot of adjusted odds ratios with 95% CIs.

The apparent AUC was 0.837. After bootstrap optimism correction, the corrected AUC was 0.799 (95% CI: 0.747–0.861), with an estimated optimism of 0.038 (Figure 3A). Calibration assessment yielded a corrected slope of 0.77, an intercept of 0.01, ICI = 0.028, E50 = 0.022, and E90 = 0.069 (Supplementary Table S3, Figure 3B), with a Brier score of 0.149. Decision curve analysis indicated positive net benefit relative to both reference strategies across the 5% to 60% threshold probability range (Figure 3C), suggesting clinical decision support value at the point of corticosteroid initiation. The corresponding nomogram is presented in Figure 2B (Supplementary Table S4). A side-by-side comparison of the performance metrics for both models is summarised in Table 3.

**Table 3:** Performance comparison of the two admission-day prediction models.

Feature	RMPP model (Cohort A)	Corticosteroid escalation model (Cohort B)
Development cohort, *n*	383	197
Outcome events, *n* (%)	106 (27.7)	61 (31.0)
Predictors retained by LASSO, *n*	9	4
Events per variable	11.8	15.2
Apparent AUC	0.865	0.837
Bootstrap optimism	0.018	0.038
Optimism-corrected AUC (95% CI)	0.847 (0.810–0.886)	0.799 (0.747–0.861)
Corrected calibration slope/intercept	0.89/0.00	0.77/0.01
ICI/E50/E90	0.016/0.014/0.038	0.028/0.022/0.069
Brier score	0.122	0.149

All metrics were obtained with the same analytical pipeline (LASSO selection, penalised logistic regression, bootstrap optimism correction with B = 2,000) applied separately to each outcome. Discrimination is reported as the area under the receiver operating characteristic curve before and after optimism correction; calibration as the corrected slope and intercept, the integrated calibration index and the median (E50) and 90th-percentile (E90) absolute calibration errors; overall accuracy as the Brier score.

AUC, area under the receiver operating characteristic curve; CI, confidence interval; CRP, C-reactive protein; E50, median absolute calibration error; E90, 90th-percentile absolute calibration error; ICI, integrated calibration index; LASSO, least absolute shrinkage and selection operator; LDH, lactate dehydrogenase; NLR, neutrophil-to-lymphocyte ratio; RMPP, refractory Mycoplasma pneumoniae pneumonia.

## 4 Discussion

Two logistic regression models were developed from routinely available admission clinical and laboratory variables to predict RMPP and corticosteroid treatment escalation, respectively. After bootstrap correction for optimism, the corrected AUC values were 0.847 and 0.799, with satisfactory calibration and positive net benefit across threshold probabilities ranging from 5% to 60% (Table 3). Both models shared a substantially overlapping predictor set, with LDH, CRP, ferritin, and NLR identified as independent predictors in each, suggesting a shared pathological basis underlying both outcomes.

The predictors identified in the RMPP model are broadly consistent with prior literature. Meta-analyses have confirmed LDH, CRP, D-dimer, and ferritin as key inflammatory biomarkers, while pleural effusion and pulmonary consolidation have been established as radiological risk factors (5, 6), and prolonged pre-admission fever duration as an independent predictor (7, 15). The corrected AUC of 0.847 attained here aligns with the discriminative estimates reported for individual biomarkers (7, 8), attained by integrating multiple routine variables. Published nomogram studies have generally reported training-set AUC values exceeding 0.90 (16–18). These estimates, however, are typically unadjusted for optimism and are likely to overestimate true model performance in independent populations. The value of 0.847 reported here is a bootstrap-corrected estimate of generalisability, methodologically distinct from those unadjusted training-set metrics, and this difference should be considered when making direct numerical comparisons (19, 20). The lower RMPP risk observed among male children in this cohort is more likely attributable to cohort composition or residual confounding than to a genuine biological sex difference, as evidence for sex-specific pathophysiology in MPP progression remains inconclusive in the existing literature.

Predictive tools for corticosteroid escalation remain sparse in the literature. A single-centre study used univariable analysis to derive cut-off values for CRP and LDH to guide pulse-dose corticosteroid decisions in children with RMPP, without consolidating these thresholds into a composite discriminative tool (21). Another investigation employed propensity score matching to examine factors associated with high-dose corticosteroid use in severe MPP, with the primary emphasis on comparative effectiveness rather than prediction model development (22). By constructing a multivariable logistic regression model for treatment escalation within the corticosteroid-treated population, the present study extends the predictive scope beyond RMPP occurrence to encompass the timing of treatment intensification, adding to the existing evidence base.

The biological relevance of these predictors is interpretable within the core pathophysiology of RMPP. Lung injury in this condition is driven predominantly by an exaggerated and dysregulated host immune-inflammatory response rather than by pathogen burden (10). CARDS toxin activates airway inflammation (23–25), Mycoplasma lipoproteins engage Toll-like receptors to promote macrophage activation and T-cell polarisation toward Th1 and Th17 phenotypes (10), and co-overexpression of CARDS toxin and tumour necrosis factor-α has been shown to predict RMPP occurrence (26). The magnitude of immune activation frequently exceeds the pathogen load, which accounts for continued clinical progression despite appropriate antibiotic therapy. Within this framework, each predictor may be regarded as a distinct dimension reflecting the degree of immune-inflammatory activation.

LDH integrates signals of tissue destruction and immune activation, making it a stable indicator of disease severity (27). CRP and ferritin reflect, respectively, the intensity of the acute inflammatory response and the degree of macrophage activation, rising proportionally to worsening inflammation. D-dimer captures the interplay between inflammation and the coagulation system and serves as an indirect indicator of endothelial injury and a hypercoagulable state (28, 29). The NLR reflects the degree of systemic immune dysregulation, while prolonged pre-admission fever duration and pleural effusion indicate, respectively, the duration of uncontrolled inflammatory activity and the extent of pulmonary and pleural involvement.

These mechanisms account for the well-established efficacy of corticosteroids in RMPP, acting through suppression of pro-inflammatory cytokine transcription and downregulation of T cell-mediated immunity (6), and for the observation that children with a greater inflammatory burden generally require more intensive immunosuppression (30). This relationship was reflected in the corticosteroid escalation model, where LDH, NLR, and ferritin demonstrated the strongest associations, consistent with clinical observations of a positive correlation between LDH and ferritin levels and the required corticosteroid dose (31). Among the four retained predictors, CRP demonstrated the weakest independent association (OR = 1.54, 95% CI: 1.02–2.34), with a near-zero LASSO-penalized coefficient, suggesting that its contribution to the escalation model is modest and may show less stability across samples. These findings suggest that biomarkers reflecting baseline inflammatory burden retain a consistent predictive direction when applied to the downstream decision of whether to intensify treatment.

### 4.1 Limitations

Several limitations warrant acknowledgement. The single-centre retrospective design precludes causal inference, and neither external nor prospective validation has been performed. The corticosteroid escalation model was derived from a treated subset of patients, introducing the possibility of treatment-indication bias, and the number of escalation events was relatively limited. The corrected calibration slope of 0.77 further indicates a degree of overfitting, reinforcing the need for prospective external calibration. The need for treatment intensification is further shaped by temporal factors including the inflammatory trajectory, macrolide resistance genotype, timing of corticosteroid initiation, and individual pharmacodynamic variability (32, 33). Single time-point admission variables cannot fully capture these dynamic processes, constraining the theoretical predictive ceiling of the model. Data collection encompassed the post-COVID-19 MPP epidemic of 2023–2024, characterised by elevated macrolide resistance rates and increased disease severity. The study population may therefore not be representative of non-epidemic MPP cohorts, limiting temporal generalisability. Corticosteroid initiation in Cohort B reflects real-world clinical decision-making influenced by unmeasured factors such as disease severity trajectories and physician practice patterns, and residual treatment-indication bias cannot be entirely excluded. Regarding diagnostic classification, a single serum antibody titre of 1:160 or greater has limited specificity given that prior infection may also yield a positive result. Institutional variation in the diagnostic definition of RMPP and in the criteria for treatment escalation may affect the generalisability of these models across centres.

### 4.2 Future work

Future work should focus on external and prospective validation in multicentre cohorts, with recalibration to the local event rate before any clinical use. Incorporating macrolide-resistance genotyping and serial biomarker trajectories may raise the predictive ceiling for treatment escalation, which single admission-day measurements cannot approach. Once validated, the nomograms could be implemented as a bedside calculator and evaluated in an impact study against usual care.

## 5 Conclusion

Two admission-day nomogram-based models were developed to support individualised risk triage in hospitalised children with MPP. The RMPP model, built on nine predictors spanning clinical, inflammatory, coagulation and radiological domains, achieved an optimism-corrected AUC of 0.847, a calibration slope of 0.89 and a Brier score of 0.122. The corticosteroid escalation model, based on four inflammatory markers, achieved corresponding values of 0.799, 0.77 and 0.149. Both conferred positive net benefit across thresholds of 5%–60%. The RMPP model was thus the better performing of the two, with higher discrimination, less optimism and closer calibration. This most plausibly reflects its complementary, non-redundant predictor set, its larger development cohort (*n* = 383 vs. 197), and the fact that escalation is a clinician-determined endpoint with an inherently lower predictive ceiling. Both nomograms require external and prospective validation with recalibration before clinical adoption.

## Data Availability

The original contributions presented in the study are included in the article/Supplementary Material, further inquiries can be directed to the corresponding author.
