# Supplementary material for: Prediction models for refractory Mycoplasma pneumoniae pneumonia and corticosteroid treatment escalation in hospitalized children: a retrospective cohort study
Source: Front Pediatr. 2026 Sep 21;14:1913556. doi: 10.3389/fped.2026.1913556 (PMC13635194; doi:10.3389/fped.2026.1913556)
Supplement: Supplementary file 1 [file Supplementaryfile1.docx]

***Supplementary Material***

1 **Supplementary Tables**

**Table S1. Baseline characteristics of corticosteroid-treated children stratified by escalation status.**

| **Characteristic** | **Overall (n=197)** | **No escalation (n=136)** | **Escalation (n=61)** | **P-value** |
| --- | --- | --- | --- | --- |
| **Demographics** | | | | |
| Age, months | 78.0 [54.0, 98.0] | 78.0 [54.0, 99.0] | 80.0 [54.0, 96.0] | 0.945 |
| Male sex, n (%) | 88 (44.7) | 62 (45.6) | 26 (42.6) | 0.816 |
| **Clinical features** | | | | |
| Fever duration, days | 6.0 [4.0, 8.0] | 5.0 [4.0, 7.0] | 7.0 [4.0, 9.0] | 0.006 |
| Illness duration, days | 7.0 [5.0, 10.0] | 7.0 [5.0, 9.0] | 9.0 [5.0, 10.0] | 0.013 |
| Peak temperature, °C | 39.2 [38.8, 39.6] | 39.1 [38.8, 39.6] | 39.3 [38.7, 39.6] | 0.611 |
| Prior macrolide, n (%) | 104 (52.8) | 73 (53.7) | 31 (50.8) | 0.828 |
| Asthma/atopy, n (%) | 36 (18.3) | 24 (17.6) | 12 (19.7) | 0.888 |
| Wheeze, n (%) | 45 (22.8) | 30 (22.1) | 15 (24.6) | 0.835 |
| Dyspnea, n (%) | 47 (23.9) | 27 (19.9) | 20 (32.8) | 0.074 |
| Oxygen at admission, n (%) | 18 (9.1) | 10 (7.4) | 8 (13.1) | 0.303 |
| **Laboratory findings** | | | | |
| WBC, G/L | 9.0 [6.8, 10.9] | 8.7 [6.7, 10.9] | 9.7 [7.2, 10.9] | 0.291 |
| Neutrophils, % | 64.6 [58.4, 71.4] | 62.5 [55.7, 69.5] | 68.4 [63.4, 77.0] | <0.001 |
| NLR | 2.3 [2.0, 3.2] | 2.2 [1.8, 2.7] | 3.2 [2.4, 4.5] | <0.001 |
| Platelet count, G/L | 302.0 [244.0, 363.0] | 299.5 [242.5, 360.2] | 307.0 [253.0, 381.0] | 0.431 |
| Hemoglobin, g/L | 119.0 [112.0, 125.0] | 120.5 [113.0, 126.2] | 117.0 [107.0, 124.0] | 0.028 |
| CRP, mg/L | 26.8 [13.8, 43.2] | 22.2 [12.7, 38.0] | 38.3 [22.1, 62.6] | <0.001 |
| Procalcitonin, ng/mL | 0.3 [0.2, 0.6] | 0.3 [0.1, 0.6] | 0.5 [0.3, 0.7] | 0.007 |
| ESR, mm/h | 38.0 [27.5, 53.5] | 36.0 [25.2, 50.0] | 44.0 [31.0, 57.0] | 0.054 |
| LDH, U/L | 387.0 [267.0, 557.0] | 344.5 [259.8, 458.5] | 587.0 [393.0, 749.0] | <0.001 |
| Ferritin, ng/mL | 168.0 [102.0, 361.0] | 144.5 [87.2, 236.2] | 318.0 [165.0, 532.0] | <0.001 |
| D-dimer, mg/L | 0.9 [0.4, 2.0] | 0.7 [0.4, 1.4] | 1.5 [0.7, 2.8] | <0.001 |
| Fibrinogen, g/L | 4.3 [3.6, 4.9] | 4.1 [3.5, 4.8] | 4.4 [3.8, 4.9] | 0.100 |
| ALT, U/L | 23.0 [11.0, 46.0] | 19.0 [9.0, 34.2] | 29.0 [17.0, 55.0] | <0.001 |
| Albumin, g/L | 39.2 [36.3, 41.8] | 39.5 [37.4, 42.4] | 37.3 [34.1, 40.3] | <0.001 |
| CK-MB, U/L | 23.0 [16.0, 30.0] | 22.0 [14.0, 30.0] | 25.0 [20.0, 30.0] | 0.061 |
| IL-6, pg/mL | 14.5 [7.6, 30.4] | 12.7 [6.1, 27.0] | 19.5 [10.6, 41.2] | 0.003 |
| **Radiological findings** | | | | |
| Consolidation (any), n (%) | 134 (68.0) | 86 (63.2) | 48 (78.7) | 0.047 |
| Consolidation ≥2/3 lobe, n (%) | 40 (20.3) | 23 (16.9) | 17 (27.9) | 0.115 |
| Pleural effusion, n (%) | 48 (24.4) | 30 (22.1) | 18 (29.5) | 0.344 |
| Atelectasis, n (%) | 25 (12.7) | 16 (11.8) | 9 (14.8) | 0.725 |

**Notes:** Summaries and tests follow Table 1. The analysis was restricted to the 197 corticosteroid-treated children with complete predictor data. All P values are two-sided.

Abbreviations: ALT, alanine aminotransferase; CK-MB, creatine kinase MB isoenzyme; CRP, C-reactive protein; ESR, erythrocyte sedimentation rate; IL-6, interleukin-6; LDH, lactate dehydrogenase; NLR, neutrophil-to-lymphocyte ratio; RMPP, refractory Mycoplasma pneumoniae pneumonia; WBC, white blood cell count.

**Table S2. Predictors retained by LASSO and their penalized coefficients.**

| **Predictor** | **Penalized coefficient (log-odds)** |
| --- | --- |
| **RMPP** | |
| Fever duration | 0.159 |
| CRP (log) | 0.433 |
| LDH (log) | 0.900 |
| D-dimer (log) | 0.294 |
| Ferritin (log) | 0.247 |
| NLR | 0.031 |
| Albumin | −0.005 |
| Male sex | −0.167 |
| Pleural effusion | 0.340 |
| **Corticosteroid escalation** | |
| CRP (log) | 0.007 |
| LDH (log) | 0.957 |
| Ferritin (log) | 0.162 |
| NLR | 0.024 |

**Notes:** Coefficients are on the log-odds scale from the L1-penalized logistic regression at the penalty selected by 5-fold cross-validation under the 1-standard-error rule. Predictors shrunk to zero are not listed.

**Abbreviations:** RMPP, refractory Mycoplasma pneumoniae pneumonia; CRP, C-reactive protein; LASSO, least absolute shrinkage and selection operator; LDH, lactate dehydrogenase; NLR, neutrophil-to-lymphocyte ratio.

**Table S3. Discrimination and calibration of the two models with internal validation.**

| **Metric** | **RMPP (whole cohort)** | **Escalation (treated subset)** |
| --- | --- | --- |
| Analytic sample, n | 383 | 197 |
| Events, n (%) | 106 (27.7) | 61 (31.0) |
| C-statistic, apparent | 0.865 | 0.837 |
| C-statistic, optimism-corrected (95% CI) | 0.847 (0.810–0.886) | 0.799 (0.747–0.861) |
| Calibration slope, corrected | 0.89 | 0.77 |
| Calibration intercept, corrected | 0.00 | 0.01 |
| ICI | 0.016 | 0.028 |
| E50 | 0.014 | 0.022 |
| E90 | 0.038 | 0.069 |
| Brier score | 0.122 | 0.149 |

**Notes:** The C-statistic and the calibration slope/intercept were corrected for optimism by bootstrap resampling (2000 replications), with the entire variable-selection and model-fitting sequence repeated in each replication; the 95% CI of the corrected C-statistic spans the 2.5th–97.5th percentiles of the resampled corrected estimates. ICI, E50 and E90 (apparent) denote the mean, median and 90th percentile of the absolute difference between predicted probability and the loess-smoothed observed risk.

**Abbreviations:** CI, confidence interval; E50/E90, 50th/90th percentile of absolute calibration error; ICI, integrated calibration index; RMPP, refractory Mycoplasma pneumoniae pneumonia.

**Table S4. Coefficients of the final logistic prediction models.**

| **Term** | **Coefficient (log-odds)** |
| --- | --- |
| **Refractory Mycoplasma pneumoniae pneumonia** | |
| Intercept | −11.6377 |
| Fever duration (days) | 0.2496 |
| log(CRP) | 0.6534 |
| log(LDH) | 1.0884 |
| log(D-dimer) | 0.4495 |
| log(ferritin) | 0.3975 |
| NLR | 0.1059 |
| Albumin | −0.0305 |
| Male sex | −0.7049 |
| Pleural effusion | 0.7327 |
| **Corticosteroid escalation** | |
| Intercept | −16.7385 |
| log(CRP) | 0.4797 |
| log(LDH) | 1.6979 |
| log(ferritin) | 0.6095 |
| NLR | 0.2575 |

**Notes:** The linear predictor is logit(p) = Σ (coefficient × value), and the predicted probability is p = 1 / (1 + e^−logit(p)). CRP, LDH, D-dimer and ferritin enter on the natural-log scale: insert the raw value in the stated unit and take its natural logarithm. Units — fever duration, days; CRP, mg/L; LDH, U/L; D-dimer, mg/L; ferritin, ng/mL; albumin, g/L; NLR, dimensionless; male sex and pleural effusion coded 1 = yes, 0 = no.

2 **Supplementary Figure**

**Figure S1.** Flowchart of patient enrollment and cohort assignment.

**Abbreviations:** CHD, congenital heart disease; GC, glucocorticoid; MPP, Mycoplasma pneumoniae pneumonia; RMPP, refractory Mycoplasma pneumoniae pneumonia.
